# Novel mutations in the *PHKB* gene in an iranian girl with severe liver involvement and glycogen storage disease type IX: a case report and review of literature

**DOI:** 10.1186/s12887-021-02648-6

**Published:** 2021-04-15

**Authors:** Zahra Beyzaei, Fatih Ezgu, Bita Geramizadeh, Alireza Alborzi, Alireza Shojazadeh

**Affiliations:** 1 Shiraz Transplant Research Center (STRC), Shiraz University of Medical Sciences, Shiraz, Iran; 2 Department of Pediatric Metabolism and Genetic, Gazi University Faculty of Medicine, Ankara, Turkey; 3 Department of Pathology, Shiraz University of Medical Sciences, Shiraz, Iran; 4 Shiraz, Iran; 5 Student Research Committee, Shiraz University of Medical Sciences, Shiraz, Iran

**Keywords:** Glycogen storage disease, Phosphorylase kinase, *PHKB*, Novel mutation, Targeted gene sequencing

## Abstract

**Background:**

Glycogen storage disease (GSD) type IXb is one of the rare variants of GSDs. It is a genetically heterogeneous metabolic disorder due to deficient hepatic phosphorylase kinase activity. Diagnosis of GSD can be difficult because of overlapping manifestations. Mutation analysis of the genes related to each type of GSD is supposed to be problem-solving, however, the presence of novel mutations can be confusing. In this case report, we will describe our experience with a young girl with the diagnosis of GSD and two novel mutations related to GSD type IXb.

**Case presentation:**

A 3-year- old girl presented with short stature, hepatomegaly, and liver cirrhosis. No specific diagnosis was made based on laboratory data, so liver biopsy and targeted-gene sequencing (TGS) were performed to find out the specific molecular basis of her disease. It was confirmed that the patient carries two novel variants in the *PHKB* gene. The variant in the *PHKB* gene was classified as pathogenic.

**Conclusions:**

This is the first reported case of a dual molecular mutation of glycogen storage disease type IXb in the same patient. Two novel variants in *PHKB* were identified and one of them was a pathogenic split-site mutation. In conclusion, for the first time, identification of the novel variants in this patient expands the molecular and the phenotype basis of dual variants in GSD-IXb.

## Background

Glycogen storage disease (GSD) type IX is caused by phosphorylase b kinase (PhK) deficiency (EC 2.7.1.38), a key enzyme in glycogen degradation [1, 2]. This enzyme is expressed in the liver and muscle tissue, though liver PhK deficiency is more common than muscle [3]. PhK is a complex enzyme including four different subunits. The α subunit is encoded by the *PHKA2* gene (MIM 306,000), namely GSD IXa, which is liver-specific with X-linked inheritance. The *PHKB* gene (MIM 261,750) indicating GSD IXb encodes the β subunit, and the *PHKG2* gene (MIM 613,027) indicating GSD IXc encodes the γ subunit [4]. Both of these subunits are autosomal recessive.

Reports on the *PHKB* gene mutations resulting in deficient phosphorylase kinase in both liver and muscle are very infrequent [5, 6]. GSD IXb is characterized by hepatomegaly, hypoglycemia, growth retardation, as well as motor developmental delays [7]. Unlike other hepatic GSDs, symptoms of GSD IXb are often mild, and patients may even become asymptomatic as they grow up [8–10].

So far, there has not been any case report of GSD type IXb from Iran. We present the first case diagnosed in our center, i.e. a child with GSD IXb, whose specific symptoms are related to a dual mutation in the *PHKB* gene.

## Case presentation

A 3-year-old girl was referred to the pediatrician with hepatomegaly and developmental delay. The girl was the first child of consanguineous marriage in an Iranian family. She was delivered following a normal and term pregnancy with a birth weight of 2.95 kg, and height of 47 cm. No hypoglycemia was noted in the perinatal period and the postnatal transition.

At the age of 1, she developed abdominal distension. However, abdominal ultrasound has been reported as unremarkable. During childhood, she frequently experienced morning nausea, vomiting, and lethargy.

At the age of 2, she was referred to our center because of developmental delay. Biochemical lab tests revealed high hepatic transaminases (ALT 375 U/L, AST 495 U/L), mild neutropenia (750 per microliter), microcytic hypochromic anemia (hemoglobin of 10.3 g/dl and hematocrit of 34.1 %, mean corpuscular volume of 73.81 fl., and low mean corpuscular hemoglobin 25.54 pg), as well as high triglyceride (498 mg/dl), and cholesterol (268 mg/dl).

**Fig. 1:**
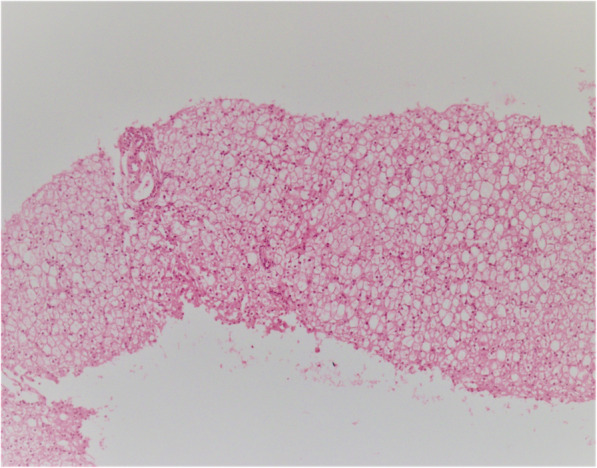
Sections from liver needle biopsy show distorted architecture with steatosis in the liver parenchyma (very similar to GSD type I)

A abdominal ultrasound revealed hepatomegaly with mild diffuse heterogeneous echogenicity of the liver parenchyma. A liver biopsy was performed, which revealed cirrhosis with severe swelling of the hepatocytes with clear cytoplasm. Portal tracts showed very mild lymphocytic infiltration (Fig. 1). According to clinical findings and liver biopsy, a hepatic form of GSD was suspected, so treatment with frequent feeds was initiated. Nevertheless, hepatic transaminase elevation persisted and ketosis with hyperlactatemia were developed. Therefore, at the age of 3, an aggressive regimen with uncooked cornstarch (5 times per day) and protein (2.5 g/kg/day) was initiated. After the diet therapy, her morning vomiting, and lethargy improved, and hepatic enzyme (ALT 80 U/L, AST 86 U/L) were decreased, but her cholesterol level increased (313 mg/dl).

Targeted gene sequencing (TGS) with a custom-targeted Ion AmpliSeq panel was performed. The panel included 7219 amplicons covering 450 genes associated with Inborn Metabolic Diseases consisting of glycogen storage disorders genes with hepatic involvement. Sanger sequencing validated identified the variants, using an ABI Prism 3500 Genetic Analyzer (Applied Biosystems, Foster City, CA, USA). Analyses were done using an Ion Torrent 540 chip (Life Technologies, Guilford, CT, South San Francisco, CA). The human genome 19 was used as the reference. Polyphen2, SIFT, and Mutation Taster were used for in silico analysis, GERP and Phastcons scores were used to evaluate the conservation of the variants. The population frequency of each variation was evaluated, using data from the gnomAD database. ACMG guidelines were used for variant interpretation [11]. The sequence variants were described according to the Human Genome Variation Society Nomenclature [12]. Interestingly, TGS findings showed that the patient carried two novel variants, which consisted of a homozygous variant c.1127-2 A > G (p.?) in exon 12, a homozygous missense variant c.2840 A > G (p.Gln947Arg) in exon 28 of the *PHKB* gene. In silico analysis revealed that the novel variant, c.1127-2 A > G (p.?) is pathogenic, which would have possibly damaged the splice site, and two other ones are variants of uncertain significance (VUS). However, samples from the parents were not available for the zygosity determination of these novel variants. It should be mentioned that both parents were asymptomatic.

## Discussion and conclusions

Herein we are reporting novel homozygous variants in the *PHKB* gene, leading to loss of function, in a 3-year-old girl born to a consanguineous family of Iranian descent.

To the best of our knowledge, our patient is the only and the first case of dual homozygote variants in GSD-IXb, with severe liver involvement. Compared with other GSD-IXb patients with a single mutation in *PHKB*, our patient who has dual variants showed a more severe short stature and liver dysfunction. To find and carefully evaluate all reported mutations and effects on the presentation of GSD IXb, we did a literature search in August 2020. To date, 23 variants were identified in *PHKB* gene in 18 patients with GSD-IXb (Table 1). A comparison of the literature showed that manifestation of GSD-IXb was highly variable, ranging from benign mild to moderate, and sometimes aggressive [2, 3, 6–10]. The age of GSD-IXb onset can be in young children with a mean age of 3.8 years. It is noteworthy that the majority of patients shown in Table 1 presented with hepatomegaly (92.85 %) and elevated hepatic transaminase (35.71 %). However, less than half of the 18 patients showed signs of hypoglycemia (27.7 %), hyperlipidemia (21.42 %), and short stature (14.28 %). Just in our case, liver cirrhosis (5.6 %) is reported.

**Table 1:** Presentation of patients and mutations in *PHKB* gene (NM_000293.1) reported in the literature

Patient	Gender	Onset (year)	Ethnicity	Hypoglycemia	Hepatomegaly	Biochemical findings	Pathological findings	Mutation	Variant type	Development	Ref.
1 2^a^ 3^a^	F M M	1.6 0.5 2.6	UK Ireland Ireland	Yes No No	No Yes Yes	NR NR NR	- Mild Liver dysfunction Slight Liver dysfunction	c.[555G > T] + [=],p.M185I c.[1257T > A]+[2336 + 965 A > C],p.Y419X c.[1257T > A]+[2336 + 965 A > C],p.Y419X	Missense Nonsense Nonsense	Normal Slightly hypotonic, speech poor Normal	[2]
1	M	2.10	German	No	Yes	NR	Slight Liver dysfunction	c.306-2 A > G	Deletion of exon 5	Normal	[3]
1 2 3 4 5	F M F M M	2.10 25 6 5 1.6	German Norway Dutch UK Israeli-Arab	Yes Just with exercise No No No	Yes Yes Yes Yes Yes	Elevated AST,ALT,TG NR Normal AST,ALT,TG NR NR	NR NR NR NR NR	c.1275dupA, p.N422KfsX c.1969 C > T p.Q657X c.1257T > A, p.Y419X c.2926G > T, p.E976X c.2896-1G > T, c.2896_2911del16 c.1285 C > T, p.R429X 7574-bp deletion of exon 8	Nonsense Nonsense Nonsense Nonsense Frame-shift deletion Nonsense Deletion of exon 8	hypoglycemic symptoms upon physical exercise Normal Normal Doll-face, abdominal extension (noted since early infancy) and muscle weakness, reduced muscle power and bulk A doll-face, mild generalized muscular hypotonia, but blood glucose and glucagon test normal	[6]
1	F	2.9	Dutch	Yes	Yes	Elevated AST, LDH, TG	No fibrosis	c.1827G > A, p.W609X IVS30^− 1^,g→t	Nonsense splice site	No hypotonia or muscle weakness	[7]
1 2 3	M M M	1.3 1.5 1.6	Canadian	No No No	Yes Yes Yes	Elevated AST,ALT Elevated AST,ALT Normal AST, ALT	NR NR NR	c.2839 C > T, p.Gln947X c.2839 C > T, p.Gln947X c.1106-2 A > G, p?	Nonsense Nonsense Split-site mu	Normal Short stature Normal	[8]
1 2 3	F M M	NR	French	No No No	Yes Yes Yes	Elevated AST,ALT Elevated AST,ALT Normal AST,ALT	Mild Liver dysfunction Slight Liver dysfunction Slight Liver dysfunction	c.1285 C > T, p.R429X c.573_577delGATTA, c.2427 + 3 A > G	Nonsense Deletion Missense	Normal Normal Normal	[9]
1	M	0.6	English	No	Yes	Normal AST,ALT	Splenomegaly, no liver biopsy	c.555G > T, p.Met185Ile c.574 A > G, p.Ile192Val	Missense Missense	Normal Normal	[10]
1	F	2	Iranian	Yes	Yes	Elevated AST,ALT, Chol	Cirrhosis	c.1127-2 A > G, p.? c.2840 A > G, p.Gln947Arg	Split site mu Missense	Short stature	Present report

^a^ Siblings

*GSD* glycogen storage disease; *FTT* failure to thrive; *TG* triglyceride; *Chol* cholesterol; *BCR* blood urea nitrogen (BUN)/creatinine ratio; *Alb* albumin; *ALT* alanine transaminase; *AST* aspartate transaminase

Molecular method confirmed a dual mutation in GSD-IXb. Co-occurrence of two different mutations in GSD subtype in one patient is exceedingly rare and has never been reported so far. Posey et al. [13] reported that out of 7374 patients only 101 (4.9 %) were diagnosed with more than one locus for the disease by performing next-generation sequencing (NGS). So, NGS is an efficient, accurate, and cost-effective method for identifying disease genes. For clinically and genetically heterogeneous diseases caused by a group of genes involving a common metabolic pathway, TGS can also be used for simultaneous sequencing of the group of candidate genes [14]. Our case demonstrates that molecular analysis especially using TGS is an essential method in the diagnosis of GSD subtypes. An early genetic diagnosis by TGS has many benefits including time and cost-effectiveness, right treatment, accurate recurrence risk advice, and where appropriate, screening of patients [15].

In conclusion, our study describes an Iranian patient who suffered from GSD-IXb. Two novel variants in the *PHKB* gene were identified, one of which is pathogen. The report of these variants could expand the mutation spectrum of GSD-IXb. Dual mutations in the GSD subtype in one patient is rare; however, with the progress in molecular diagnostic methods, we may be able to identify more patients with multiple mutations in different genes, and because of that, our knowledge about inherited human diseases will be improved.

## Data Availability

The datasets used and/or analyzed during the current study are available from the corresponding author on reasonable request.

## Abbreviations

ALT: : Alanine transaminase
AST: Aspartate transaminase
GSD: Glycogen storage disease
NGS: Next-generation sequencing
PAS: Periodic acid–Schiff
PhK: Phosphorylase b kinase
TGS: Targeted Gene Sequencing
VUS: Variants of uncertain significance
